# Both J- and L-shaped upper hemisternotomy approaches are suitable for total arch replacement with frozen elephant trunk in patients with Type A dissection

**DOI:** 10.3389/fcvm.2022.998139

**Published:** 2022-11-10

**Authors:** Zhonglu Yang, Hui Jiang, Yu Liu, Yuguang Ge, Huishan Wang

**Affiliations:** Department of Cardiovascular Surgery, General Hospital of Northern Theater Command, Shenyang, China

**Keywords:** aortic dissection, total arch replacement, minimal invasive incision, hemisternotomy, frozen elephant trunk

## Abstract

**Background:**

Minimally invasive total arch replacement (TAR) with frozen elephant trunk (FET) implantation can be carried out through J-, L-, and inverted T-shaped upper ministernotomy. L- and inverted T-shaped upper ministernotomy are selected mostly for their better surgical view compared to J-shaped. However, few studies have paid attention to the difference in clinical effects between J- and L-shaped upper hemisternotomy in acute Type A aortic dissection (ATAAD).

**Materials and methods:**

We retrospectively analyzed 74 consecutive patients with ATAAD who underwent TAR with FET implantation between December 2019 and October 2020. Patients were divided into the L group (*n* = 31, L-shaped upper hemisternotomy) and the J group (*n* = 43, J-shaped upper hemisternotomy). Perioperative characteristics were recorded.

**Results:**

No significant difference was found in any of the pre-operative, post-operative, or follow-up variables between the two groups. However, the CPB establishment time in the J group was significantly shorter than that in the L group (65.0 ± 17.9 min vs. 77.9 ± 17.2 min, *P* < 0.05). Other intraoperative variables showed no significant difference.

**Conclusion:**

Total arch replacement with frozen elephant trunk implantation is feasible and can be carried out safely through J-shaped or L-shaped incision. A J-shaped incision might be beneficial for single incision, while an L-shaped incision might be beneficial if an extra incision is required to achieve better artery perfusion.

## Introduction

Acute Type A aortic dissection (ATAAD) is a lethal disease that requires prompt diagnosis and surgical intervention (1). The frozen elephant trunk (FET) procedure facilitates distal aortic anastomosis during total arch replacement (TAR) (2), which is a widely used procedure in China with a low prevalence of morbidity and mortality (3, 4). However, all four studies mentioned employed full sternotomy.

Recently, minimally invasive approaches in cardiovascular surgery have led to faster recovery, enhanced thoracic stability, and less pain (5, 6). Consequently, the use of ministernotomy for aortic surgery is becoming more widespread, especially for surgery of the aortic valve, aortic root, and ascending aorta (7, 8). However, minimally invasive approaches are seldom performed in the treatment of ATAAD. Previously, we demonstrated the benefits of J-shaped upper hemisternotomy in ATAAD recovery (9, 10). However, previous studies on minimally invasive aortic procedures were mostly carried out through L-shaped or inverted T-shaped upper ministernotomy. Specifically, inverted T-shaped upper ministernotomy is considered to provide better surgical view compared to J-shaped (11, 12). Few studies have paid attention to the difference between J- and L-shaped upper hemisternotomy in ATAAD.

Here, we retrospectively analyze 74 patients with ATAAD who underwent surgery for TAR with FET through single (J- or L-shaped) hemisternotomy at our cardiovascular surgery center, aiming to advance the development of minimally invasive aortic surgery.

## Materials and methods

### Patient characteristics

We retrospectively collected data of 74 patients diagnosed with ATAAD based on CT angiography (CTA) who underwent TAR with FET between December 2019 and October 2020. Based on the type of hemisternotomy, these patients were divided into the L group (upper-left hemisternotomy approach, *n* = 31) and the J group (upper-right hemisternotomy, *n* = 43). Operation in the J group was carried out between December 2019 and May 2020, while operation in the L group was carried out between June 2020 and October 2020. The main inclusion criterion was diagnosis of ATAAD involving the aortic arch. Exclusion criteria included neurologic complications including cerebral infarction and cerebral hemorrhage, malperfusion syndrome, and concomitant operations that required full sternotomy (such as coronary heart disease, mitral valve disease, and congenital heart disease). The study was approved by the Ethics Committee of the General Hospital of Northern Theater Command, Shenyang City, China [K(2020)19]. All patients provided informed consent.

### Surgical procedure

**FIGURE 1:**
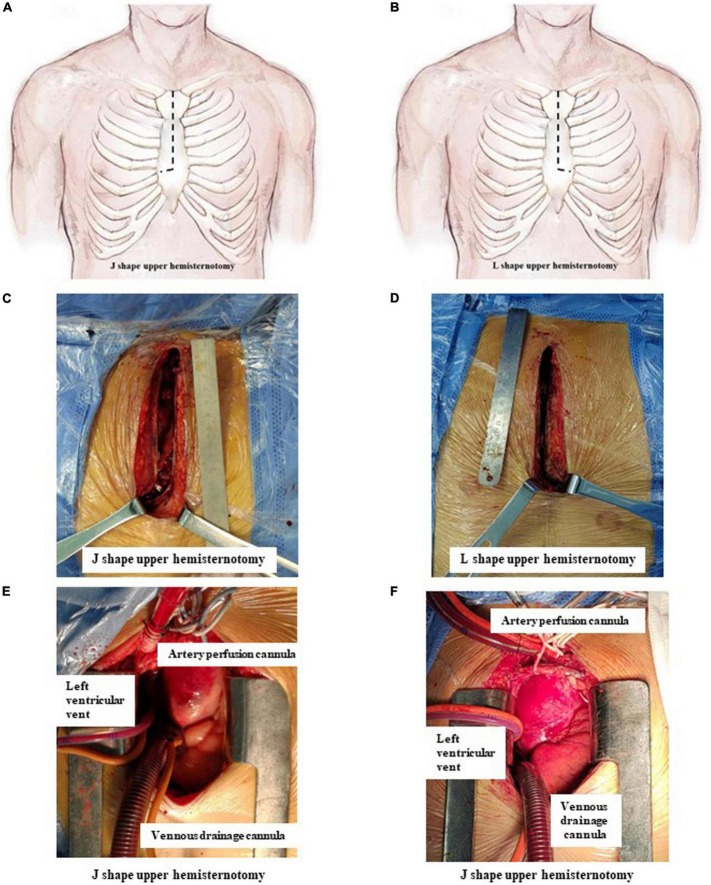
J- and L-shaped upper hemisternotomy. **(A,C)** J-shaped upper hemisternotomy is opened from the sternal notch to the fourth intercostal space and then extended to the right fourth intercostal space. **(B,D)** L-shaped upper hemisternotomy is opened from the sternal notch to the fourth intercostal space and then extended to the left fourth intercostal space. **(E)** Cannulation finished *via* J-shaped upper hemisternotomy. **(F)** Cannulation finished *via* L shape upper hemisternotomy.

Surgery in both groups was performed as described in our previous study (10). Briefly, a single incision from the sternal notch to the level of the fourth intercostal space was made and then extended to the right fourth intercostal space (J group, Figures 1A,C) or the left fourth intercostal space (L group, Figures 1B,D). Other surgical procedures were the same in the two groups.

The strategy of cannulation in cardiopulmonary bypass (CPB) was based on surgical exploration. Direct innominate artery cannulation was mainly selected for artery perfusion, and right atrial cannulation was selected for venous drainage. When the innominate artery was dissected, the right subclavian artery and the right or left common carotid artery were used as alternative cannulation sites. Blood cardioplegia was used for myocardial protection. The right superior pulmonary vein was cannulated as left ventricular vent (Figures 1E,F).

Moderate hypothermia circulatory arrest was carried out after aortic root procedures were completed and the systemic temperature cooled to 28–30°C. Cerebral perfusion was achieved through bilateral selective antegrade cerebral perfusion (bSACP) as previously reported (9, 10). Near-infrared spectroscopy (NIRS) monitoring was used for cerebral protection. Circulatory arrest was conducted after occlusion of the innominate artery, and the FET was placed as previously reported (10, 13). Briefly, after arch arteries were transected and the proximal segment was sutured, a stent graft (MicroPort Medical Co. Ltd., Shanghai, China) was inserted into the true lumen of the distal aorta in a bound, compressed state after the distal aorta was transected between the origin of the left subclavian artery (LSCA) and the left carotid artery. Then, a four-branch prosthetic graft (Vascutek Ltd., Terumo Aortic, Inchinnan, Scotland, United Kingdom) was firmly attached to the distal aorta. After anastomosis was completed, lower body perfusion was recovered through the four-branch prosthetic graft. To decrease the duration of SACP and the cross-clamp time, the left common carotid artery, proximal aortic stump, LSCA, and innominate artery were successively anastomosed to the prosthetic graft. All patients in both groups received a temporary pacing wire to the right ventricle and one drainage tube for pericardial draining.

### Definitions for complications

Permanent neurological deficit (PND) was defined as previously described. Post-operative renal dysfunction was defined as a creatinine level of >230 mM (twice the normal value). Perioperative blood transfusion was defined as intraoperative and/or post-operative transfusion of red blood cells, fresh frozen plasma, and/or platelets. CPB establishment time was calculated from skin incision to the start of CPB.

### Statistical analysis

Data were retrospectively collected. Continuous variables are described as mean ± standard deviation or as median (interquartile range), and categorical variables are described as frequency (%). Data were analyzed using SPSS Version 20.0 (SPSS Inc., Chicago, IL, USA). A Chi-squared test or Fisher’s exact test was used to compare the distribution of categorical variables between groups. Continuous variables were analyzed using Student’s *t*-test or the Wilcoxon rank sum test. A two-sided *P*-value of less than 0.05 was considered to indicate a significant difference.

## Results

The pre-operative data of the 74 patients are presented in Table 1. Patients were divided into the J group (*n* = 43) and the L group (*n* = 31) based on the different incisions. There was no significant difference in baseline characteristics between groups.

**TABLE 1:** Baseline demographics.

Clinical variables	J group (*n* = 43)	L group (*n* = 31)	*P*-value
Age (Years)	52.4 ± 10.2	48.4 ± 12.1	0.130
Male, *n* (%)	30 (69.8)	22 (71.0)	0.911
Body Weight (kg)	76.2 ± 15.4	79.4 ± 13.2	0.370
BMI (%)	26.0 (24.2, 28.7)	26.1 (24.1, 28.5)	0.891
LVEF (%)	59.1 ± 3.0	57.7 ± 3.2	0.253
Smoking, *n* (%)	20 (46.5)	18 (58.1)	0.327
Diabetes, *n* (%)	2 (4.7)	0	0.223
Hypertension, *n* (%)	32 (74.4)	22 (71.0)	0.742
Bicuspid aortic valve, *n* (%)	0	1 (3.2)	0.193
Marfan’s syndrome, *n* (%)	3 (7.0)	3 (9.7)	0.675
Onset time (d)	3 (2, 4)	2 (1, 4)	0.113

BMI, body mass index; LVEF, left ventricular ejection fraction.

Intraoperative variables are listed in Table 2. No difference in surgical procedures, arterial perfusion position, CPB time (163.3 ± 34.4 min vs. 174.5 ± 35.3 min, *P* = 0.177), cross-clamp time (98.3 ± 23.8 min vs. 96.6 ± 27.0 min, *P* = 0.776), and circulatory arrest time (16.4 ± 16.6 min vs. 22.7 ± 19.0 min, *P* = 0.142) was observed between the J and L groups. The CPB establishment time in the J group was significantly shorter than that in the L group (65.0 ± 17.9 min vs. 77.9 ± 17.2 min, *P* = 0.003).

**TABLE 2:** Intraoperative data of patients.

Clinical variables	J group (*n* = 43)	L group (*n* = 31)	*P*-value
CPB time (min)	163.3 ± 34.4	174.5 ± 35.3	0.177
CPB establishment time (min)	65.0 ± 17.9	77.9 ± 17.2	0.003
Cross-clamp time (min)	98.3 ± 23.8	96.6 ± 27.0	0.776
Circulatory arrest time (min)	16.4 ± 16.6	22.7 ± 19.0	0.142
Minimum nasopharyngeal temperature (°C)	29.3 ± 1.4	29.0 ± 1.7	0.534
Arterial perfusion position			
Innominate artery, *n* (%)	30 (69.8)	20 (64.5)	0.823
Right subclavian artery, *n* (%)	1 (2.3)	1 (3.2)	0.814
Right common carotid artery, *n* (%)	1 (2.3)	0	0.393
Left common carotid artery, *n* (%)	2 (4.7)	2 (6.5)	0.710
Surgical procedures			
Aortic valvuloplasty, *n* (%)	10 (23.3)	6 (19.4)	0.688
Aortic valve replacement, *n* (%)	1 (2.3)	1 (3.2)	0.814
Bentall procedure, *n* (%)	7 (23.3)	7 (22.6)	0.495
Total arch replacement + FET [*n* (%)]	43 (100.0)	31 (100.0)	1.000
ECMO [*n* (%)]	0	1 (3.2)	0.193

CPB, cardiopulmonary bypass; FET, frozen elephant trunk; ECMO, extracorporeal membrane oxygenation.

The post-operative variables are shown in Table 3. In total, six patients died (four in the J group and two in the L group) after the operation (9.3% vs. 6.5%, *P* = 0.658) due to post-operative massive cerebral infarction (3), multiple organ failure (1), sudden hemodynamic changes (1), and malignant arrhythmia (1). There was no difference between groups in ventilation time, intensive care unit (ICU) stay, first 24 h chest tube drainage, intraoperative or post-operative transfusion, reventilation, PND, acute renal failure, post-operative length of stay, and hospital costs.

**TABLE 3:** Post-operative data of patients.

Clinical variables	J group (*n* = 43)	L group (*n* = 31)	*P*-value
Ventilation time, (h)	35.2 ± 31.5	40.2 ± 50.0	0.610
ICU stay, (h)	51.3 ± 38.8	55.2 ± 53.7	0.718
First 24 h chest tube drainage, (ml)	235.2 ± 142.5	224.6 ± 115.2	0.772
Transfusion, *n* (%)	23 (76.7)	16 (51.6)	0.873
Reventilation, *n* (%)	2 (4.7)	1 (2.5)	0.223
PND, *n* (%)	4 (13.3)	0	0.081
Acute renal failure, *n* (%)	2 (4.7)	4 (12.9)	0.077
In-hospital death, *n* (%)	4 (9.3)	2 (6.5)	0.658
Post-operative length of stay	15 (13–20)	17 (11.5–21)	0.713
Hospital costs (10,000 CNY)	20.3 (18.7–22.2)	21.0 (17.9–24.6)	0.463
Follow-up time (months)	30 (28, 31)	24 (22, 26)	0.000
Follow-up rate (%)	43 (100)	31 (100)	
Late death, *n* (%)	5 (12.8)	3 (10.3)	0.754

PND, permanent neurological deficit; CNY, Chinese Yuan.

The number of follow-up patients were 39 in J group and 29 in L group.

After surgery, 39 patients in the J group and 29 patients in the L group were followed-up with. The median follow-up time was 30 (28, 31) months in the J group and 24 (22, 26) months in the L group (*P* < 0.001; this difference is caused by the different start time points for follow-up between the two groups). During follow-up, eight patients died (five patients in the J group and three patients in the L group); the mortality rate was not significantly different between the two groups (12.8 vs. 10.3, *P* = 0.754) (Table 3).

## Discussion

Upper hemisternotomy has been shown to be safe and effective in aortic valve surgery. It has the advantages of fast post-operative recovery, short ventilation time, short ICU stay, little blood transfusion, and little incision pain (14, 15). However, few studies on TAR surgery through a UHS have been reported. Ahmad et al. carried out TAR with FET through L-shaped hemisternotomy with artery perfusion in the right axillary artery (12). Inoue et al. reported T-shaped hemisternotomy instead of L-shaped hemisternotomy as a standard incision procedure (11). Staromłyński et al. reported hemi-arch replacement through V-shaped hemisternotomy with an incision of only 6 cm (16).

Our study included 74 patients who required TAR with FET, which was performed through a single UHS in which the sternum was incised in a J shape or an L shape from the sternal notch down to the fourth intercostal space. In both J-shaped and L-shaped hemisternotomy, TAR with FET can be successfully carried out through a single incision if the surgeon has enough experience in total arch surgery through full sternotomy, aortic valve replacement, and the Bentall approach through upper hemisternotomy. Based on our experience, there are some differences between J-shaped and L-shaped TAR with FET through a single upper hemisternotomy approach.

First, the J-shaped incision provides better exposure of the outline of the aortic valve field, the surrounding tissue, and the right ventricular outflow tract, which is beneficial for innominate artery cannulation, right atrial cannulation, and left ventricular vent cannulation; the L-shaped incision provides poor exposure of the brachiocephalic trunk artery and the right atrium, and hence, it is more difficult to make the incision for cannulating the right upper pulmonary vein and atrium. Specifically, in some patients with Marfan’s syndrome or with severe dilatation of the ascending aorta, the right atrium is compressed by the aorta, which may impede exposure of the operating field, forcing the surgeon to perform vent cannulation through the pulmonary artery or to canulate the right upper pulmonary vein after CPB. When the trunk of the brachiocephalic artery is damaged by dissection, cannulation should be performed in the right common carotid artery or the right subclavian artery, which makes artery perfusion canulation more difficult. Therefore, previous studies selected the right axillary artery or one of the femoral arteries for artery perfusion (11, 12), avoiding the innominate artery cannulation, but adding the need for another incision. Second, the exposure of the left subclavian artery was poor after J-shaped incision, which makes LSCA anastomosis to the prosthetic graft difficult. In our experience, anastomosis could be facilitated using an elastic occlusion band instead of an occlusion clamp to occlude LSCA, combined with reasonable pulling by an assistant. L-shaped incision provides better exposure of the LSCA, facilitating anastomosis. However, care must be taken not to damage the left internal mammal artery, which might affect future coronary artery bypass surgery. Third, when the pericardium is lifted after thoracotomy, the L-shaped incision leads to a smaller deviation of the heart toward the right side of the chest compared with J-shaped incision, which may have less influence on the circulatory hemodynamics and lead to a smaller fluctuation of arterial blood pressure. After the L-shaped incision, it is easier to place a pacemaker on the epicardial surface than after a J-shaped incision.

In the present study, only CPB establishment time was significantly longer in the L group than in the J group (65.0 ± 17.9 min vs. 77.9 ± 17.2 min, *P* = 0.003). This might be because the surgeon was more experienced in making J-shaped incisions than L-shaped incisions (9, 10). However, other intraoperative characteristics and follow-up variables showed no difference between the two groups, which might indicate that with enough practice, J-shaped incision and L-shaped incision will result in the same clinical effects. All results demonstrated that J-shaped and L-shaped incisions are both safe and feasible for TAR with FET. Moreover, J-shaped incision might be more suitable for single incision with innominate artery cannulation, and L-shaped incision might be more suitable if a second incision with cannulation on the right axillary artery or one of the femoral arteries is required.

## Limitations

There are some limitations in the present study. First, the study has a time bias. Second, this is a retrospective study, which may cause selection bias. Third, the study is a single-center study with small sample size, which might cause confounder bias. All these limitations should be avoided in future studies through improving sample size, using randomized controlled trials, and using long-term follow-up.

## Conclusion

Total arch replacement with frozen elephant trunk implantation is feasible and can be carried out safely through J-shaped incision and L-shaped incision. A J-shaped incision might be more suitable for single incisions, while an L-shaped incision might be more suitable if an extra incision to improve artery perfusion is necessary.

## Data availability statement

The raw data supporting the conclusions of this article will be made available by the authors, without undue reservation.

## Ethics statement

The studies involving human participants were reviewed and approved by the Ethics Committee of General Hospital of Northern Theater Command. The patients/participants provided their written informed consent to participate in this study. Written informed consent was obtained from the individual(s) for the publication of any potentially identifiable images or data included in this article.

## Author contributions

ZY and YL drafted the manuscript and collected the data. HJ and YG carried out the surgeries. HW provided guidance for this study. ZY acquired and analyzed the data and carried out the surgeries. All authors contributed to the manuscript and approved the submitted version.
